# A multidisciplinary approach to inform assisted migration of the restricted rainforest tree, *Fontainea rostrata*

**DOI:** 10.1371/journal.pone.0210560

**Published:** 2019-01-25

**Authors:** Gabriel C. Conroy, Yoko Shimizu-Kimura, Robert W. Lamont, Steven M. Ogbourne

**Affiliations:** Genecology Research Centre, University of the Sunshine Coast, Maroochydore, Queensland, Australia; Instituto Nacional de Investigacion y Tecnologia Agraria y Alimentaria, SPAIN

## Abstract

Assisted migration can aid in the conservation of narrowly endemic species affected by habitat loss, fragmentation and climate change. Here, we employ a multidisciplinary approach by examining the population genetic structure of a threatened, dioecious rainforest tree of the subtropical notophyll vine forests of eastern Australia, *Fontainea rostrata*, and its potential requirements for population enhancement and translocation to withstand the effects of anthropogenic fragmentation and climate change. We used microsatellite markers to gain an understanding of the way genetic diversity is partitioned within and among the nine extant populations of *F*. *rostrata* identified in this study. We combined the results with species distribution modelling to identify populations vulnerable to possible future range shifts based on climate change projections. We found regional differences between the species’ main distribution in the south and a disjunct northern population cluster (*F*_*RT*_ = 0.074, *F*_*SR*_ = 0.088, *F*_*ST*_ = 0.155), in mean allelic richness (*A*_*R*_ = 2.77 vs 2.33, *p* < 0.05), expected heterozygosity (*H*_*E*_ = 0.376 vs 0.328), and inbreeding (*F* = 0.116 vs 0.219). Species distribution models predicted that while southern populations of *F*. *rostrata* are likely to persist for the next 50 years under the RCP6.0 climate change scenario, with potential for a small-scale expansion to the south-east, the more highly inbred and less genetically diverse northern populations will come under increasing pressure to expand southwards as habitat suitability declines. Given the species’ genetic structure and with the aim to enhance genetic diversity and maximise the likelihood of reproductive success, we recommend that plant reintroductions to supplement existing populations should be prioritised over translocation of the species to new sites. However, future conservation efforts should be directed at translocation to establish new sites to increase population connectivity, focussing particularly on habitat areas identified as persisting under conditions of climate change.

## Introduction

The subtropical rainforest communities of eastern Australia have been significantly reduced and highly fragmented following intensive land clearing for agriculture and urban development, leading to decreased population sizes and increased population isolation for many species [1]. Taxa confined to these remnants are likely to become increasingly vulnerable to extinction in the future through the loss of genetic diversity over time due to limited gene flow, genetic drift and inbreeding [2, 3]. In addition, the effects of climate change are predicted to further compromise the ability of many species to persist, due to potential range shift pressures and phenological modifications affecting population dynamics and genetic structure [4, 5]. Locally endemic species in rainforest communities are considered particularly vulnerable because of narrow thermal tolerances, relatively small population sizes, limited dispersal and restricted distribution [6].

In Australia, the climatic fluctuations of the Plio-Pleistocene have led to significant changes in the distributions of rainforest communities [7, 8]. Climatically stable areas that persisted throughout the Quaternary are known to have acted as refugia for genetic and taxonomic diversity, often containing endemic species with limited dispersal capabilities [8–10]. A recent study identified five centres of endemism in southeast Queensland and northern New South Wales, including the region between the Sunshine and Fraser Coasts, which may have functioned as a refuge for subtropical rainforest species during periods of alternating climate [11]. While some species survived by dispersing through suitable habitat, others persisted *in-situ* or became extinct [12]. Current patterns of gene flow are often an indicator of the migration potential of a species, and detection of low levels of gene flow may indicate a limited ability to keep pace with range shifts [13]. Rossetto *et al*. [8] suggest that high plant endemism in Australian subtropical rainforests may be due to dispersal limitations, rather than bottlenecks or habitat specificity. Hence the probability of endemic species with limited dispersal capabilities persisting under ongoing habitat fragmentation and climate change may depend on a taxon’s adaptive potential or ability to keep pace with a changing environment through emigration [14].

**Fig 1:**
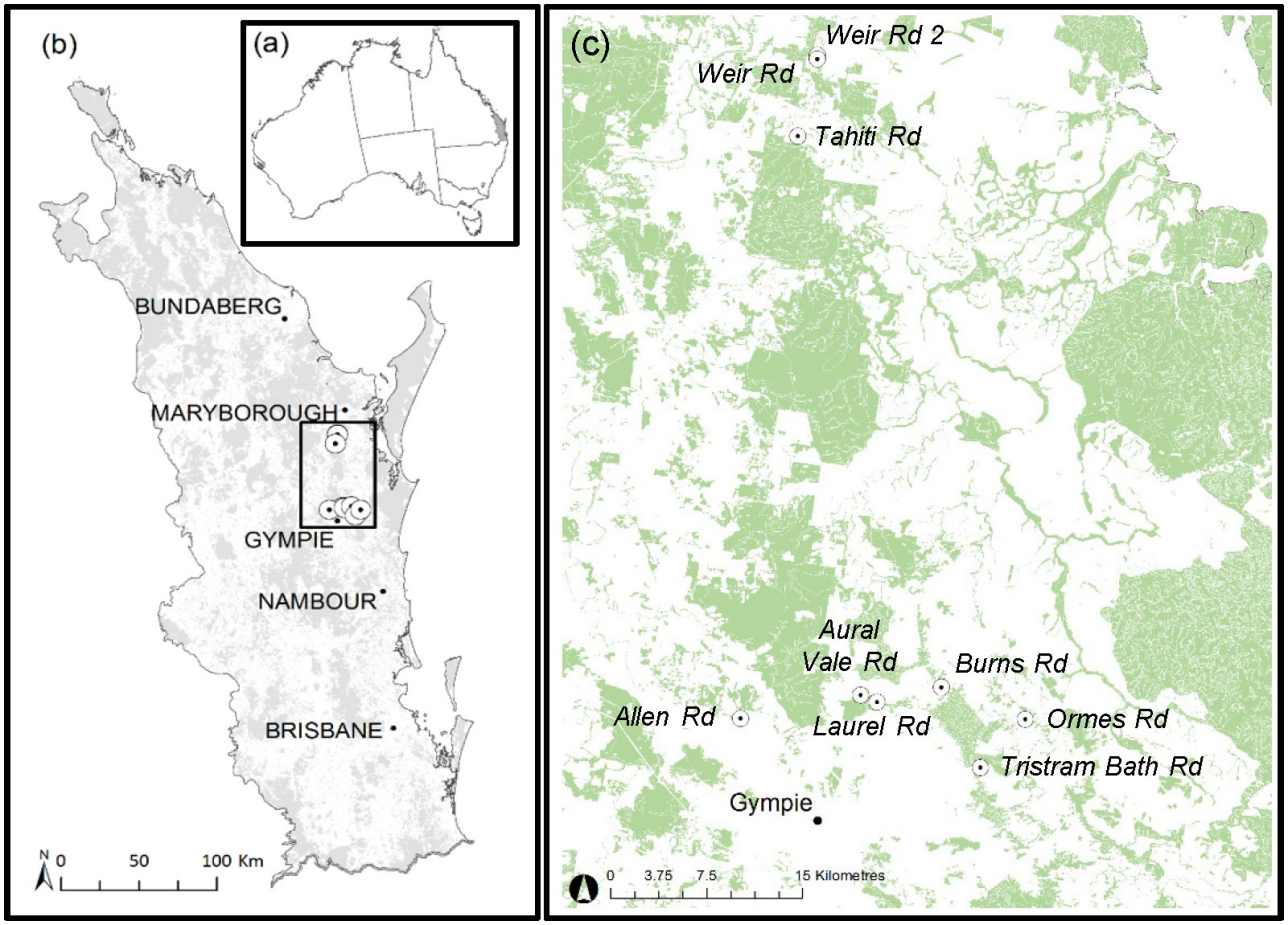
Map of the study area showing (a) its position within southeast Queensland, (b) population locations and remnant vegetation [20] in southeast Queensland, and (c) Population locations and remnant vegetation of the northern and southern sampling sites in greater detail.

*Fontainea rostrata* Jessup & Guymer (Euphorbiaceae) is a dioecious tree known from only a small number of the remaining fragmented pockets of subtropical rainforests of the Burnett-Mary region of southeast Queensland. These remnants occur across a latitudinal range of approximately 80 km, between Maryborough and Gympie (Fig 1). Individuals reach ~9 m in height and are predominantly found in notophyll vine forests growing along river terraces or gullies [15, 16]. The species is thought to be insect-pollinated with female plants producing fruit up to 3 cm in diameter, which are primarily dispersed by gravity a short distance from the parent tree. As a result, populations typically occur over a relatively small area, made up of a limited number of discrete clumps of differently-aged juvenile cohorts, often found around one or a few adult trees. Secondary dispersal via hydrochory, where conditions are favourable, particularly along drainage lines, is probably responsible for population establishment over greater distances [15, 16]. However, despite repeated searches over several decades, no populations, or even individuals of *F*. *rostrata*, have been found growing along the 45 km of steep-sided banks fringing Tinana Creek, the northward-flowing waterway linking the southeast and northern population clusters (Fig 1). As most records are relatively dated, current population sizes are unknown [17], and the species is currently listed as vulnerable under the Australian government’s *Environment Protection and Biodiversity Conservation Act 1999* [18] and the *Queensland Nature Conservation Act 1992* [19]. Threats include extensive loss and fragmentation of habitat due to clearing for agriculture and plantation forestry, with ongoing degradation arising from anthropogenic interruptions to landscape scale genetic processes, weed invasion, and the incursion of fire into remnant patches [17].

In recent years, there has been increasing interest in assisted migration and reintroduction of threatened species as a conservation measure in response to habitat loss, fragmentation and climate change [21]. Conservation reintroduction strategies range from low-risk *in situ* population reinforcement of current populations, to high-risk *ex situ* translocation beyond the species’ historical range [21, 22]. Whilst it is ideal to select target sites with protected tenure that provide ecologically suitable habitat within a species’ current distribution [23, 24], the location and extent of suitable habitat for many plant species is expected to shift under the conditions associated with projected climate change [25, 26].

In addition to environmental and ecological suitability, the genetic structure of source populations is considered useful in guiding the successful reintroduction of threatened plant species [27]. For instance, translocated populations are likely to go through genetic bottlenecks, the effects of which may be more pronounced if founder plants are sourced from small, isolated populations with low genetic diversity, leading to negative demographic and genetic consequences [3, 27]. Conversely, mixing genetically dissimilar founder stock from multiple populations with different ecological histories could potentially introduce outbreeding depression within reintroduced populations [28]. However, if used appropriately, knowledge of a species’ population genetic structure, has the capacity to enhance the viability of some of its more genetically depauperate populations through the genetic rescue effect [22, 29]. The importance of determining genetic structure to guide conservation efforts is well recognised [28] and a growing number of studies have modelled the effects of climate change on numerous taxa around the world [30–32]. The combination of field assessment, molecular genetics and habitat modelling is however an underutilised, yet pragmatic approach, to evaluate conservation and reintroduction strategies, as well as gauging their success.

In this study, we employed a multidisciplinary approach to determine whether assisted migration is a suitable and necessary conservation management strategy for *F*. *rostrata*. Specifically, we determined whether the combined assessment of genetic diversity, genetic structure, and species distribution modelling (SDM) to classify current and future habitat suitability under projected climate change, can effectively identify and evaluate the potential of translocation and enhancement strategies in the mitigation of threats currently facing *F*. *rostrata*’s more vulnerable populations. As the species occurs in a variety of rainforest types comprising several hundred taxa, findings are likely to be of broader relevance to species sharing similar life-history characteristics, while the multidisciplinary methodology reported here provides a novel benchmark for conservation management.

## Methods

### Field sampling and genetic analysis

We conducted surveys at 26 sites of suitable habitat, including all previously known locations as well as intervening riparian corridors connecting the northern and southern population clusters near Maryborough and northeast of Gympie, respectively (Fig 1). Nine populations of *F*. *rostrata* were identified from which a total of 211 individuals covering the geographical range of the species were sampled (Fig 1). Populations were named after access points into small, but variably-sized remnants of notophyll vine scrub of between approximately 2 and 28 hectares (mean ~12 ha) in size (with one larger remnant of ~300 ha, encompassing Laurel Rd and Aural Vale Rd). Leaf tissue was collected from 20–29 individuals per population, and total genomic DNA was extracted from silica-dried leaf tissue using the DNeasy Plant Mini Kit (Qiagen, Hilden, Germany) following the manufacturer’s instructions.

Twelve polymorphic microsatellite loci (Table 1) with consistent PCR amplification and clear electrophoretic signatures were selected to assess population genetic variation. A detailed description of marker development using GS-FLX Titanium chemistry (Roche Applied Science; Mannheim, Germany) is given in Agostini *et al*. [33]. PCR was undertaken as per Lamont *et al*. [34], with DNA products separated by capillary electrophoresis on an AB 3500 Genetic Analyser (Applied Biosystems). Fragment sizes were determined relative to an internal lane standard (GS-600 LIZ; Applied Biosystems) using GENEMARKER version 2.4.0 (SoftGenetics, State College PA, USA) [35] and double-checked manually. Individuals with low or missing peaks were amplified and genotyped a second time.

**Table 1:** Characterisation of microsatellite loci in 211 individuals of *Fontainea rostrata*.

Locus GenBank	Repeat motif	Primer sequences (5’-3’)	Size range (bp)	*PIC*	*N*_*A*_	*H*_*O*_	*H*_*E*_	*F*
FP20 KY406156	(GAGTTT)4	F: GCAAGTTCCAGGCACTGTTT R: ACTCACATCCAAATGCACCA	149–155	0.028	2	0.028	0.028	-0.130
FP21 KC759358	(TA)_13_	F: TCACTGAATTCGCTTGGTTG R: TGCAAATACCAGAAGTGCCA	184–238	0.508	4	0.379	0.570	0.149
FP24	(TA)_10_	F: GGATGACAAAATTCCTTGCC	213–239	0.566	9	0.336	0.621	0.287
KY406157		R: TCCATGTTATTAGCAGCACCA						
FP32 KC759359	(GT)_8_	F: CTGGCTTGCATTTGCTTGTA R: TGCTAAACTTCAAGGGCTTAGG	182–192	0.064	4	0.052	0.065	0.192
FP33	(AG)_7_	F: GAAGCGAAGGAAAATCAGCA	165–171	0.339	4	0.114	0.406	0.686
KY406158		R: GCAATACAGCAAGCCAATCA						
FP38	(GAAGAG)_6_	F: ATGAAGTTATTGCAAGGGCG	137–143	0.365	2	0.355	0.481	0.128
KY406159		R: TCCTGTAGGGTTGTCTTCCG						
FP39 KC759362	(GA)_15_	F: CTGCACGACAAGAAAACTCG R: TGAGTCAATATTGTAAGGGAATTATGA	189–207	0.512	4	0.427	0.575	0.203
FP40 KC759363	(TG)_16_	F: TTCTCGTCCTCTACTGGGCT R: CCCTACCTTTCCCACTCACA	132–146	0.501	7	0.227	0.544	0.572
FP41	(CT)_9_	F: TTGCACCGTTAAAGCATTTG	131–155	0.330	2	0.393	0.418	-0.016
KY406160		R: GATTCCAATCAACCAGTTCCA						
FP44 KC759364	(AT)_7_	F: TGAAGCTAATTGCTTGATCTTCC R: GGGTATTTATTTTCTTGTTTGTTTCC	108–114	0.253	4	0.213	0.268	0.114
FP49 KM213753	(GA)_8_	F: TTTATACAACCACCAGTCGCC R: CACCTTCACTGAAATTCTCTTCTTC	163–169	0.333	4	0.441	0.368	-0.295
FP64 KM213757	(GAC)_11_	F: ACGGTGAAGACGATGATGGT R: CGTGTGTTACCTCTTCTTCAGC	99–123	0.678	7	0.749	0.721	-0.235
			**Mean**	0.373	4.417	0.310	0.422	0.138

*PIC*—polymorphic information content; *N*_*A*_—number of alleles; *H*_*O*_—observed heterozygosity; *H*_*E*_—expected heterozygosity; *F*—inbreeding coefficient.

GenAlEx version 6.5 [36] was used to generate allelic frequencies and determine genetic diversity parameters including the mean number of alleles per locus (*A*), observed heterozygosity (*H*_*O*_), expected heterozygosity under conditions of Hardy-Weinberg equilibrium (*H*_*E*_), and the fixation index (*F*) as a measure of past inbreeding [37]. Polymorphic information content (*PIC*) was calculated in CERVUS version 3.0.3 [38]. Measures of allelic richness (*A*_*R*_) and private allelic richness (*PA*_*R*_) for each population were obtained via rarefaction using the program HP-RARE [39], based on a minimum sample size of 20. BOTTLENECK [39] was used to test for the likelihood of recent deviations from mutation-drift equilibrium. The intermediate two-phased model (TPM) was selected due to its suitability for microsatellite data [40, 41], with deviations from equilibrium determined using Wilcoxon’s Sign Rank tests. SPSS (IBM version 24) was used to test for significant differences between population clusters for genetic diversity and inbreeding measures. Independent t-tests were applied for all parameters, except for private allelic richness (*P*_*AR*_), where a Mann-Whitney U-test was used due to a skewed distribution.

We used several methods to analyse population structure across *F*. *rostrata*’s distribution. The average pair-wise level of genetic differentiation (*F*_*ST*_) [37] among populations and between the northern and southern regions was calculated using multilocus comparisons based on 999 permutations in GenAlEx version 6.5 [36]. To look for further evidence of genetically differentiated groups of populations, we ran the Bayesian genetic clustering algorithm in STRUCTURE version 2.3.4 [42]. Correlated allele frequencies were applied using an admixture model, and ten independent runs for each value of *K* (number of clusters) between 2 and 9 were performed, employing a burn-in of 100,000 followed by 500,000 Markov Chain Monte Carlo (MCMC) steps for each run. The geographic location of samples was omitted from the cluster analysis, with results across each run summarised to infer the optimal value of *K* [43] as implemented in STRUCTURE HARVESTER web version 0.6.93 [44], processed using CLUMPP version 1.1.2 [45], and visualised with DISTRUCT version 1.1 [46]. Nei’s unbiased genetic distance [47] was used to generate a genetic distance matrix as the basis of an analysis of molecular variance (AMOVA) to determine genetic relationships within and among populations and the regions identified with the STRUCTURE analysis using GenAlEx version 6.5 [36]. Mantel’s test [48] was run in GenAlEx 6.5 [36], to examine whether a relationship between genetic and geographic distances existed across the species distribution.

### Species distribution modelling

The study area for the SDM component was the southeast Queensland region. A total of 20 reliable historical and current records of *F*. *rostrata* presence were compiled from herbarium voucher records [49], online database records [50] and field census records from this study (S1 Table). All current records were ground-truthed and confirmed during the field census. We selected 17 variables as potential candidate predictors for the *F*. *rostrata* model (S2 Table). Fourteen bioclimatic GIS data layers of southeast Queensland representing the current climatic conditions were derived from SimCLIM v 3.0.0.5 [51]. SimCLIM is a computer-based climate simulation database system which generates temperature and precipitation anomalies from up to 40 global circulation models (GCM) [52] with spatial resolutions ranging from 100 to 400 km horizontal grids, downscaled to a 250 m × 250 m grid resolution for the southeast Queensland region. Future climate surfaces can be generated for four emission scenarios based on the IPCC 5th assessment report (AR5) [53] at yearly intervals up to 2100, using a 1990 baseline climate generated from the interpolation of long-term average monthly weather data. Environmental substrate variables such as soil and geology are known to be critical factors to define plant distributions and have a large impact on their persistence [54]. The digital atlas of Australian soils dataset at a map scale of 1:2,000,000 [55] (S3 Table) and the detailed solid geology of Queensland dataset at a map scale of 1:50,000 [56] (S4 Table) were also used as candidate predictors in the model. In order to factor in proximity to watercourse as one of the potential model predictors, the Queensland drainage dataset at a map scale of 1:25,000 [57] was used to calculate Euclidean distance matrices. These were re-sampled using a 250 m digital elevation model [51] prior to modelling analysis in ArcGIS version 10.2 [58].

We used MaxEnt v3.3.3k [59–61]to model the habitat distribution of *F*. *rostrata* under current and future environmental conditions. MaxEnt is a software program for modelling species distributions using presence-only data on the principle of a maximum entropy algorithm [62] Whilst there are other methods available for modelling species distributions such as generalised linear models (GLMs), generalised additive models (GAMs), mechanical models and ensemble techniques, we used MaxEnt because it has been widely applied and used by government agencies and research institutions for modelling plant distributions under both current and future environments [63–67] and has been shown to perform well in comparison to other models where relatively few presence records are available [68]. To identify the most informative contributing subsets of variables, Spearman’s rank correlation [69] and MaxEnt jack-knife tests were conducted to identify significantly correlated pairs of variables (*r* > 0.80), whereupon the variables that made the least contribution to model performance were omitted. The final baseline model was run with three-fold cross validation. Ten thousand background points were randomly selected by the model from the study region and the model was run with 500 iterations using the logistic output format, which represents the habitat suitability probability values within the range of 0–1 for each grid cell in the model [61].

Two future climate projections were derived using SimCLIM v 3.0.0.5 [51] for one future point (2065) and two emission scenarios (AR5 RCP4.5 and RCP6.0) using an ensemble of 40 GCMs currently available in SIMCLIM (S5 Table), whereby the different members of an ensemble are automatically averaged together to provide an estimate of the climate change projections [52]. The use of GCM model ensembles has been thought to filter out individual model bias and reduce inherent uncertainties among GCMs [70]. The RCP scenario storylines project a wide range of possible changes in greenhouse gas (GHG) emissions into the future due to anthropogenic activities [53]. RCP4.5 scenario is an intermediate scenario which assumes that GHG emissions will peak in the mid-21^st^ century, prior to stabilising shortly after 2100. The RCP6.0 scenario is also an intermediate scenario, with the GHG emissions peaking in the late-21^st^ century then stabilising [71, 72], meaning both RCP choices are in alignment with the aims of identifying general trends in climate change induced changes in habitat suitability. In order to explore a range of potential uncertainties associated with projections of future climate surfaces, sensitivity analyses were conducted for each bioclimatic variable by generating 10^th^, 50^th^ and 90^th^ percentile projections. The 50^th^ percentile climate projection surfaces were ultimately used for the final model. An assumption was made that the geological/geomorphological variables would remain constant for the entire modelling period of 50 years, thus current condition datasets were used for all projections.

Model performance was evaluated by the area under the curve (AUC) in receiver operating characteristic analysis (ROC) of the cross validated model output. An AUC score of 1.0 indicates a statistically valid, perfect model fitting, while an AUC value of <0.5 indicates a model performing poorly and no better than random [60]. The baseline model output was used to determine which variables were the best predictors of the species habitat distribution. The model projections indicate analogous future habitat distribution of the species for both the RCP4.5 and RCP6.0 scenarios, thus results are only presented for the latter. Model outputs were reclassified to enable the discrimination of high probability habitat for *F*. *rostrata* by applying the minimum habitat suitability threshold value based on the mean 10^th^ percentile training presence value across cross-validated models, which uses the 10th percentile of the probability threshold range of the species presence records [73]. The reclassified baseline model outputs and the regional ecosystem (RE) vegetation dataset of Queensland v 8.0 [74] were then overlaid to determine in which rainforest community types *F*. *rostrata* currently occurs, and to assess the extent of vegetation in each community type within high probability habitat areas using ArcGIS version 10.2 [58].

## Results

### Genetic diversity and inbreeding

A total of 53 alleles were resolved across the 12 microsatellite loci assayed in 211 *F*. *rostrata* individuals with a mean number of alleles per locus at the species level (*N*_*A*_) of 4.417 (Table 1). The number of alleles per locus ranged from 2 to 9 with *PIC* values between 0.028 to 0.678 (mean *PIC* = 0.373; Table 1). The mean number of alleles per locus (*A*) within respective populations was 2.657 (Table 2), although following correction for population size, mean allelic richness per locus (*A*_*R*_) was 2.622 (Table 2). However, when analysed in terms of the six southern (Allen Rd, Aural Vale Rd, Laurel Rd and Ormes Rd, Burns Rd, Tristram Bath Rd) versus the three northern populations (Tahiti Rd, Weir Rd and Weir Rd 2), the downstream northern population cluster was found to be significantly less genetically diverse than the southern population cluster when corrected for population sample size using rarefaction (*A*_*R*_ = 2.33 vs 2.77, *p* < 0.05).

**Table 2:** Summary of genetic measures for the nine populations (and northern and southern population clusters) of *Fontainea rostrata*.

Population	*n*	*A*	*A*_*R*_	*PA*_*R*_	*H*_*O*_	*H*_*E*_	*F*
Allen Rd—S	23	2.417	2.37	0.06	0.286	0.348	0.225
Aural Vale Rd—S	21	2.917	2.90	0.00	0.389	0.396	0.013
Laurel Rd—S	24	3.083	3.03	0.00	0.326	0.398	0.178
Ormes Rd—S	20	2.833	2.83	0.00	0.283	0.333	0.194
Burns Rd—S	22	2.833	2.79	0.00	0.432	0.426	-0.025
Tristram Bath Rd—S	26	2.750	2.70	0.17	0.327	0.358	0.110
Tahiti Rd—N	29	2.500	2.41	0.00	0.247	0.354	0.269
Weir Rd—N	24	2.167	2.16	0.00	0.264	0.307	0.206
Weir Rd 2—N	22	2.417	2.41	0.17	0.254	0.324	0.183
**Mean** SE	**23.44** (0.250)	**2.657** (0.124)	**2.622** (0.095)	**0.044** (0.025)	**0.312** (0.025)	**0.360** (0.022)	**0.148** (0.037)
Southern Cluster	136	2.806	2.77*	0.04	0.341	0.376	0.116
Northern Cluster	75	2.361	2.33*	0.06	0.255	0.328	0.219

*n*—number of plants sampled per population; *A*—mean number of alleles per locus; *A*_*R*_—allelic richness (based on a minimal sample size of 20); *PA*_*R*_—private allelic richness; *H*_*O*_—mean observed heterozygosity; *H*_*E*_—mean expected heterozygosity; *F*—fixation index.

Six private alleles were observed in this study, with frequencies ranging between 0.022 and 0.136 (S8 Table; Fig 1) giving a mean private allelic richness per population (*PA*_*R*_) of 0.044 (Table 2). While most rare alleles were detected in only a single individual from each site, both Tristram Bath Rd and Weir Rd 2 (S8 Table) possessed population-specific alleles, which occurred in approximately 25% of the population.

The observed heterozygosity (*H*_*O*_) across populations ranged from 0.247 to 0.432 (mean *H*_*O*_ = 0.312), with levels of expected heterozygosity (*H*_*E*_) calculated under conditions of Hardy-Weinberg Equilibrium between 0.307 and 0.426 (mean *H*_*E*_ = 0.360) (Table 2). Once again, the northern population cluster displayed lower values of diversity (*H*_*O*_ = 0.255; *H*_*E*_ = 0.328) compared to the southern populations (*H*_*O*_ = 0.341, *p > 0*.*05*; *H*_*E*_ = 0.0.377, *p > 0*.*05*) (Table 2). The fixation index (*F*) indicated a moderate level of inbreeding (*F* = 0.148) when averaged across all populations of the species, with individual population values ranging from -0.025 to 0.269 (Table 2). However, when separated into the southern and northern populations, the southern populations were less inbred (*F* = 0.116) than the northern populations (*F* = 0.219; *p > 0*.*05*). These trends were confirmed via three sub-analyses where three separate sets of three loci (e.g. *FP20*, *FP32*, *FP33* –low PIC; *FP20*, *FP38*, *FP41* –low *N*_*A*_; *FP24*, *FP33*, *FP40*—out of trend *F*) were removed from the analysis (S9 Table). The mean number of alleles per locus (*A*), observed heterozygosity (*H*_*O*_) and expected heterozygosity (*H*_*E*_) were consistently higher in the southern versus northern populations, while the inbreeding values were consistently lower in the southern populations (Table 2). Together with the regional differences in allelic diversity and heterozygosity, this suggests that the northern populations have established from a depauperate subset of the main population cluster higher up in the Tinana Creek catchment via hydrochorous dispersal and have lost diversity through inbreeding and random fixation of alleles due to founder effects. This is consistent with the findings of the Mantel’s test, which identified a small (*Rxy* = 0.174) but significant (*p* = 0.010) correlation between genetic and geographic distances over the species range indicating a genetic cline between distributional extremes (S1 Fig). The BOTTLENECK analysis did not detect a recent signature of an excess (*p* > 0.05) of either homo- or heterozygotes at any of the loci tested, suggesting the downstream populations have been established for a considerable time.

### Population genetic structure and gene flow

The mean pair-wise level of genetic differentiation (*F*_*ST*_) between populations was 0.155, translating to a low level of historical gene flow, barely sufficient to combat genetic drift; Table 3). A regional AMOVA further divided population dissimilarity based on a 7% difference (*F*_*RT*_ = 0.074) between the three population clusters, with another 8% (*F*_*SR*_ = 0.088) of genetic variation due to differences among populations within regions. An additional 14% of variation was due to differences between individuals, leaving 71% of the genetic variation contained within individuals within populations (S2 Fig). Pair-wise population *F*_*ST*_ values were all significantly different from zero (*p* < 0.001) and ranged from a minimal level of differentiation between two relatively proximate populations (Tahiti Rd and Weir Rd 2, *F*_*ST*_ = 0.034; Table 3) to negligible contact between two geographically isolated populations (i.e. Allen Rd and Ormes Rd; *F*_*ST*_ = 0.214; Table 3).

**Table 3:** Pairwise population *F*_*ST*_ (below diagonal).

	Allen Rd	Aural Vale Rd	Laurel Rd	Ormes Rd	Burns Rd	Tristram Bath Rd	Tahiti Rd	Weir Rd	Weir Rd 2
Allen Rd	0.000								
Aural Vale Rd	0.071	0.000							
Laurel Rd	0.111	0.115	0.000						
Ormes Rd	0.214	0.124	0.127	0.000					
Burns Rd	0.142	0.148	0.141	0.125	0.000				
Tristram Bath Rd	0.160	0.143	0.151	0.077	0.095	0.000			
Tahiti Rd	0.160	0.148	0.138	0.167	0.133	0.199	0.000		
Weir Rd	0.156	0.168	0.107	0.143	0.148	0.197	0.034	0.000	
Weir Rd 2	0.135	0.166	0.132	0.205	0.169	0.151	0.097	0.057	0.000

*F*_*ST*_—genetic variance contained within a subpopulation relative to total genetic variance.

**Fig 2:**
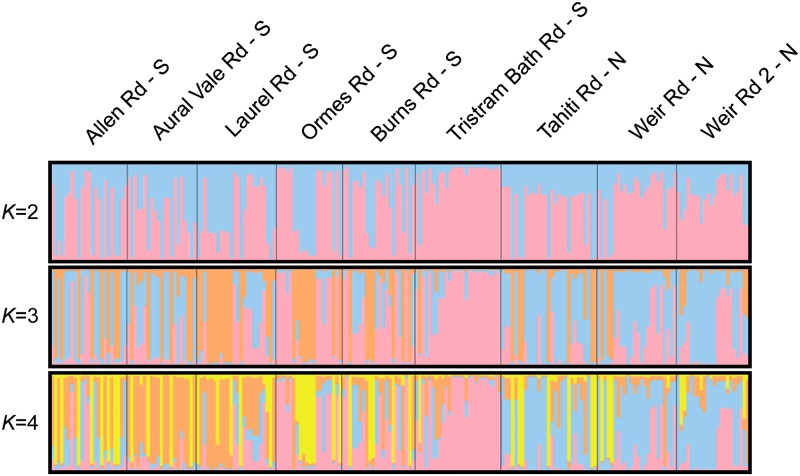
STRUCTURE barplots for values of K = 2–4 showing the genetic relationships between populations, within and among regions.

**Fig 3:**
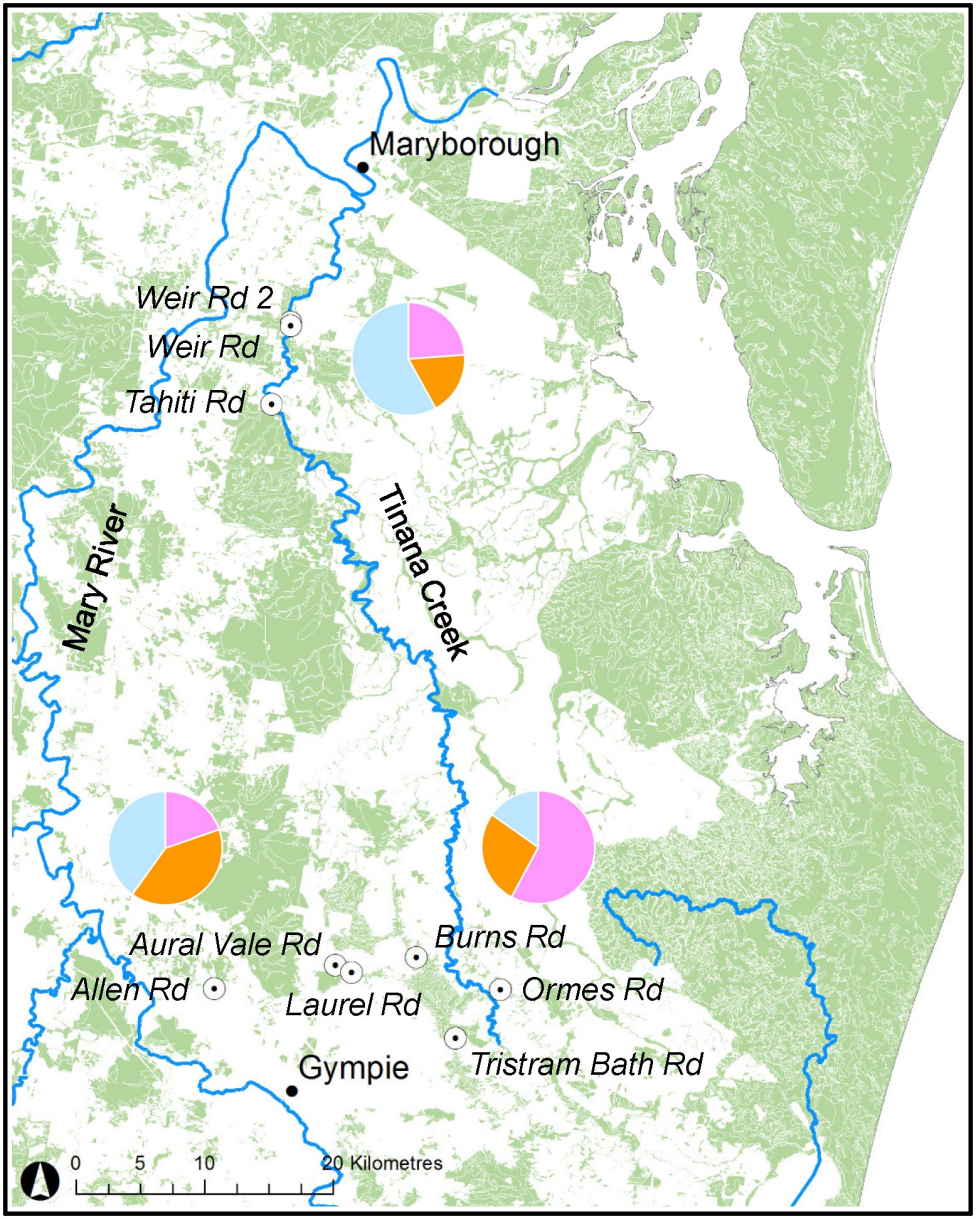
The average membership of individuals of the K = 3 clusters for each population cluster are presented as pie charts, superimposed onto the location map, including shaded remnant vegetation [20], to provide geographic perspective.

The STRUCTURE analysis (Fig 2) identified three genetic groups within the nine populations, clustering based on geographic proximity and segregating the two more elevated, mesic southern groups from the three populations of the drier northern, s using multilocus genotypes *(K* = 3) providing membership proportions to each value of *K* among regions (Fig 3; S3 Fig). However, to avoid underestimating the intricacies of *F*. *rostrata*’s population structure [66], values *K* = 2–4 are shown (Fig 2).

### Species distribution under current and future climatic conditions

**Fig 4:**
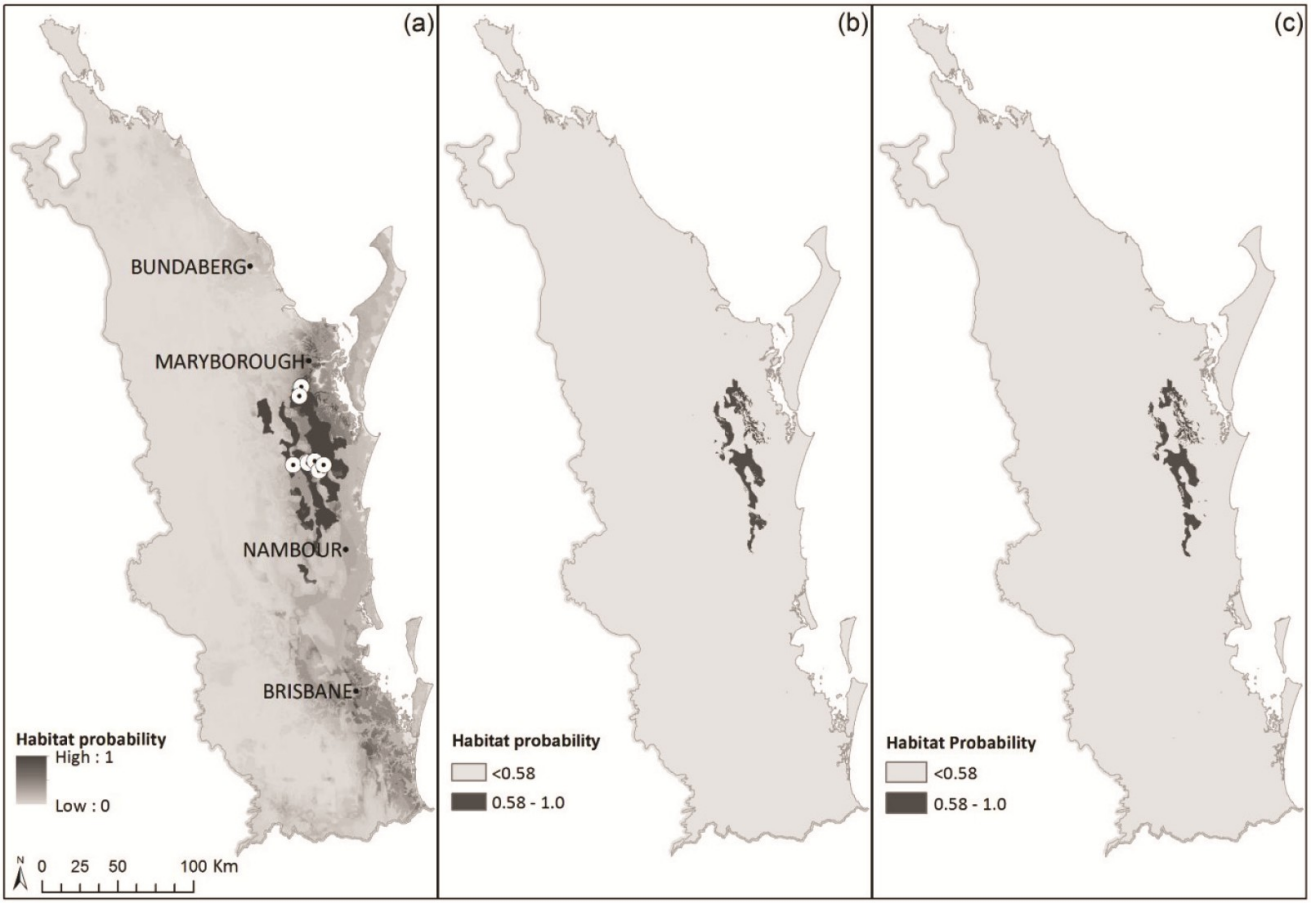
(a) Habitat suitability probability map, with studied populations indicated with white circles, and (b) binary map showing clear discrimination of high and low probability habitat under current environmental conditions, and (c) binary map of year 2065 with RCP6.0 scenario (AR5) within the southeast Queensland region.

Six predictor variables were selected for the final *F*. *rostrata* model (AUC = 0.954; S6 Table). The three variables that made the highest relative contribution to the model were soil (62.1%), precipitation of the coldest quarter (29.5%) and geology (5.9%). The model predicted patchy habitat distribution for *F*. *rostrata* across southeast Queensland, with the highest concentration of suitable habitat found in the central part of the region (Fig 4a). The binary map indicated that high probability habitat areas are currently found from south of Maryborough to north of Nambour (Fig 4b), covering a total area of approximately 865 km^2^ (S7 Table). The nine known populations of *F*. *rostrata* are found in seven subtropical rainforest RE types, two of which are classified as endangered and one of which is ‘of concern’ (*Vegetation Management Act*, 1999 (VMA); S7 Table). Only 8% of the high-quality habitat is contained within these RE types, with another 30% in other RE types where *F*. *rostrata* has not been previously reported, leaving approximately 62% of ‘high-quality habitat’, which currently exists as non-remnant, degraded areas (S7 Table).

The models predicted southward range shift in the habitat distribution of *F*. *rostrata* within southeast Queensland over the next 50 years, with habitat areas located in the north of the species’ range predicted to become less suitable by 2065. Conversely, the species’ high probability habitat areas in the central and southern part of the region are predicted to remain intact for the next 50 years, with the potential habitat range predicted to expand to the south-east thereafter (Fig 4c).

## Discussion

### Genetic diversity and inbreeding

Many Australian rainforest endemics belonging to ancient Gondwanan lineages, such as *Fontainea*, have small, isolated populations confined to refugia spread across a limited geographic distribution, due to long-term contractions arising from environmental changes associated with the glacial cycles of the late Quaternary [7, 8, 34, 75]. According to population genetics theory, taxa surviving under such conditions will generally display low levels of genetic diversity due to the increased homozygosity resulting from inbreeding among related individuals [2, 76, 77]. This theory was supported by the findings of Rossetto et al. [78], who used chloroplast genomic data from 71 rainforest species and found rapidly expanding lineages of more recent Indo-Malesian origin to be characterised by significantly lower levels of genetic diversity [79] than refugial Gondwanan lineages with longer local histories. However, this is not always the case as species with restricted distributions and poor dispersal capabilities can be less genetically diverse than widespread species [80–82]. For instance, *Macadamia tetraphylla* (*H*_*E*_ = 0.512), from the rainforests of northern New South Wales is comprised of 12 populations totalling 350 individuals and has a small distributional range of less than 100 km [83], whereas more widely distributed taxa, such as the Gondwanan species *Ocotea catharinensis* (*H*_*E*_ = 0.730) and *O*. *odorifera* (*H*_*E*_ = 0.782) from Brazil’s Atlantic Rainforest with a range of 800–1000 km were found to display correspondingly higher levels of diversity [84]. Similarly, the level of microsatellite diversity resolved across populations of *F*. *rostrata* (mean *H*_*E*_ = 0.360) was somewhat depauperate in comparison to the widespread rainforest tree, *Toona ciliata* var. *pubescens* (*H*_*E*_ = 0.644) [85], but was similar to that found in a tropical congener, *F*. *picrosperma* (*H*_*E*_ = 0.407) [34]. While estimates of genetic diversity (*H*_*E*_) [86] are necessarily dependent on the polymorphic information content (*PIC*) of the loci employed, the microsatellite markers used for this study were the product of rigorous screening, with approximately 75 monomorphic primer pairs (or 80% of the initial 454-derived loci tested) being rejected because their non-informative nature [33] and therefore an *H*_*E*_ of 0.360 is likely an appropriate representation of the genetic diversity for *F*. *rostrata*.

*Fontainea rostrata* is a dioecious, obligate outbreeder, although relative to other outbreeding genera such as *Eucalyptus* (*E*. *grandis H*_*E*_ = 0.863 [87]; *E*. *nitens H*_*E*_ = 0.911 [88]; *E*. *globulus H*_*E*_ = 0.851 [89]), the species has low levels of genetic diversity. In *F*. *picrosperma*, low levels of diversity were attributed to cycles of contraction and recolonization triggered by climatic fluctuations resulting in genetic bottlenecks and the species’ habit of forming clumps of related individuals within populations, due to both the poor dispersal capabilities of propagules [34] and pollen limitation [90]. Similar mechanisms have likely contributed to the low diversity found in *F*. *rostrata* populations, which is significant as we found 62% of the species’ core habitat has already been substantially modified by clearing for agriculture, grazing and plantation forestry. Rossetto *et al*. [91] note that rainforest contraction in Australia led to the extinction of key seed dispersers of large-fruited rainforest taxa, such as the southern cassowary (*Casuarius casuarius johnsonii*), with potential detrimental effects to the genetic integrity of populations of many large-seeded species. In fact, they suggest that the loss of such dispersal agents may have had a greater influence on the distributions and population genetic structure of susceptible taxa than decreased habitat availability. Given the current lack of dispersal agents, it is probable that habitat fragmentation and degradation since European settlement may be exacerbating adverse genetic and demographic effects in *F*. *rostrata* populations through the accelerated loss of connectivity among populations, decreasing gene flow and increasing the risk of inbreeding depression and localised extinctions in the future, particularly in the less diverse and more highly inbred northern populations (*F* = 0.239). In contrast, the combined (historical) fixation indices for the two southern populations was low (*F* = 0.029), a level similar to that recorded for widespread eucalypt species (*F* = 0.016) [87–89], although this may well rise as the number of generations post-isolation of remnants containing *F*. *rostrata* continues to increase.

### Population structure and gene flow

This study found evidence of genetic differentiation (*F*_*IT*_ = 0.291; S2 Fig) between the two geographically distinct population clusters, which combined, constitute the genetic structure of *F*. *rostrata* at the species level. Such information can be used to optimise strategies to cover the predicted effects of climate change. Short-distance seed dispersal in *F*. *rostrata* is primarily by gravity, however hydrochory provides a means of secondary dispersal over greater distances. Both processes are apparent in the clumped population structure of the species not only within populations, but also at a landscape level.

The northern population cluster of Tahiti Rd, Weir Rd and Weir Rd 2 are between 45–60 km downstream of the southern population cluster, in the lower reaches of Tinana Creek, some 22 km before its confluence with the Mary River near Maryborough. Hence, these two population clusters are possibly related historically via hydrochorous dispersal. However, as one population from each region possessed a private allele at relatively high frequency across the individuals genotyped, the fact that the habitat supporting the northern cluster could have been isolated as long ago as the climatic fluctuations of the Quaternary is possible.

The southern population cluster can be further divided into south-eastern (Burns Rd, Ormes Rd & Tristram Bath Rd; Tinana Creek Catchment) and south-western (Aural Vale Rd, Allen Rd & Laurel Rd; Mary River Catchment) clusters based on watershed characteristics (Fig 3); an observation that is supported by the STRUCTURE analysis (Fig 2). The south-western populations are situated along first and second order streams flowing west into the Mary River, north of Gympie. These populations are topographically separated by approximately 10 km from the south-eastern population cluster of Ormes Rd, Burns Rd and Tristram Bath Rd, which are associated with gullies draining eastwards into the upper reaches of Tinana Creek. Although historical genetic differentiation estimates between the two southern population clusters were relatively high, it is possible that these two groups were more connected via ‘ghost’ populations, prior to European settlement. However, under current conditions, and particularly considering the findings of Grant et al. [90] regarding pollen limitation and hence fruit-set in the genus, it is unlikely that effective gene flow is still occurring.

In its more elevated upper reaches (150–200 m asl), Tinana Creek is steep-sided and fast-flowing when in flood. Any rainforest species that manage to recruit on the terraces between flood events appear to be mostly removed by the next flow. It is only on the lower elevation floodplains (50 m asl), now extensively cleared for the cultivation of sugar cane, that flow rates decrease enough to allow more permanent establishment of propagules from upstream. In fact, despite a considerable search effort spanning two decades (including surveys not part of this study), no populations or even individuals of *F*. *rostrata* have been found along Tinana Creek between the south-eastern and northern population clusters. Therefore, with suitable habitat predicted to move southwards under the climatic conditions predicted over the coming century, management of the three isolated northern populations at Tahiti Rd, Weir Rd and Weir Rd 2 should be prioritised regarding assisted migration and/or population enhancement.

### Current and future habitat distributions of *F*. *rostrata* in southeast Queensland

Many narrowly endemic rainforest species, such as *F*. *rostrata*, are known to exhibit high habitat specificity and narrow thermal tolerances [6]. However, the likelihood of persistence in geographically-restricted species under conditions of ongoing habitat fragmentation and climate change will be largely dependent on their phenotypic plasticity and/or emigration and dispersal ability [14]. Populations of *F*. *rostrata* are currently confined to seven subtropical rainforest community types, which comprise only 8% of the high-probability habitat under present environmental conditions, indicating the potential vulnerability of the species’ and its core habitat into the near future. Added to this, under RCP6.0 conditions, a relatively moderate southward range shift of *F*. *rostrata* was predicted by the model, with high-probability areas in the northern region projected to contract southwards into new areas of suitable habitat predicted to emerge between the current northern and southern population clusters, and to the south of the existing southern populations. However, the unassisted migration of *F*. *rostrata* propagules may not be an option due to extensive habitat-loss, -fragmentation and -degradation of these areas over the last 150 years, not to mention the absence of actual physical avenues for hydrochorous dispersal from source populations. The species’ medium to large-sized seeds, and possibly the seeds of co-occurring taxa with similar biology and life histories, will therefore need to be manually introduced into suitable pockets of remaining habitat as predicted by the SDM.

In this study SDM provided a useful tool to evaluate general trends in habitat distribution, as required for the aims of this study. However, it does not always represent the uncertainty associated with model selection, variable selection and future climate projections [92]. For instance, we used only MaxEnt models to project the distribution of *F*. *rostrata* in this study, and the predictive power of our models could be further improved by exploring several other modelling algorithms to assess and compare the full amount of model predictive uncertainties. Additionally, a relatively low number of presence records exist for *F*. *rostrata*, and our SDM was restricted to the southeast Queensland region, which may not fully represent the species theoretical niche. However, *F*. *rostrata* is a highly endemic species confined to small, fragmented habitat remnants within the modelled region, and despite extensive field surveys to bolster existing historical occurrence records, the species only occurs in relatively few locations, even within suitable habitat. Thus, as with other studies that utilise a relatively low number of occurrence records by necessity [63–67], MaxEnt was a suitable choice to identify trends and inform conservation management decisions for the species.

Another important consideration when appraising model efficacy is that climate projections are subject to varying levels of uncertainty dependent on the combination of downscaling methods and choice of GCMs and emission scenarios [70]. The future climate projection layers from GCMs are generally statistically downscaled to reach finer spatial resolutions using climate simulation software such as SimClim [51] and these may result in pseudoreplication. In this study, geological and geomorphological variables were coupled with climatic variables, however, coupling fine-resolution regional scale substrates and low-resolution global scale climate may result in models weighted toward the utilised substrate variables. Although uncertainties arising from the GCMs could have been explored individually, an ensemble of 40 GCMs was used to average individual model uncertainties at the time of the climate projection modelling. This is in alignment with our study aims of identifying general trends for climate change induced changes in habitat suitability and/or range shifts, and in accordance with our choice of intermediate RCP scenarios. Thus, despite these constraints, the results of this study demonstrate the utility of SDMs in evaluating general trends regarding the habitat distribution of *F*. *rostrata* at a regional scale under current and future climatic conditions. This approach gives advanced warning of the conservation and genetic management actions required for *F*. *rostrata*, and perhaps similar restricted endemic taxa, over the next 50 years and beyond.

### Genetics, habitat, climate and conservation planning

Maintenance of genetic diversity is considered an essential determinant of long-term persistence for many threatened plant species [76, 93]. Although there are modest levels of genetic differentiation between northern and southern populations of *F*. *rostrata*, likely due to differences in multilocus composition arising from unidirectional hydrochorous dispersal and founder effects within the downstream (northern) populations, overall allelic diversity is relatively limited. Therefore, considering the low availability of suitable habitat within appropriate ecosystems, set amongst a landscape matrix that generally comprises non-remnant, degraded land, the implementation of a genetic rescue program to increase local genetic diversity, reduce inbreeding and potentially improve reproductive success is recommended. While the use of neutral markers means that low genetic diversity does not automatically equate to reduced population fitness, and in some instances less genetically diverse populations may be better adapted to local environmental conditions, the impacts of climate change and continuing degradation of landscape scale ecological processes via anthropogenic causes means that the adaptive potential for *F*. *rostrata* is likely to be compromised. Mixing genetic stock through population enhancement has the potential to increase adaptive capacity, maintain species-level genetic structure [94] and enhance population viability in the long-term [93, 95] particularly given the predicted range shifts within *F*. *rostrata*’s core habitat as a result of climate change.

Although there is potential for hybridization, genetic swamping, and outbreeding depression when mixing genetically dissimilar stocks [28], the levels of genetic diversity in *F*. *rostrata* populations are sufficiently low that the species would likely benefit from a controlled program of genetic supplementation among populations, as a means of increasing allelic diversity and heterozygosity, and mitigating further inbreeding [96]. The SDM model predicted that current populations of *F*. *rostrata* are likely to persist for the next 50 years under the RCP6.0 climate change scenarios. This suggests that the consolidation of existing populations should be prioritized over translocation of the species to newly established sites, with reintroduction efforts focusing on increasing population sizes, and increasing allelic diversity, especially in the more isolated populations, as these are most likely to suffer the deleterious effects associated with genetic erosion. Translocation efforts in this manner should be in cognizance of local adaptations being important to the survival of low diversity populations, and of the fact that in some instances, populations with low measured diversity may be well adapted to localised conditions and hence provide valuable genetic source material [97, 98]. Translocations to areas containing the specific subtropical rainforest communities identified in this study (S7 Table), that are also south of the areas predicted to be affected by a range shift should then become the goal of ongoing conservation efforts, as these areas are likely to have a better chance of providing climatically and environmentally stable conditions given future climate change predictions.

This project validates the importance of a multidisciplinary approach to guide conservation management of restricted, threatened taxa. We have demonstrated the value of combining SDMs alongside an understanding of genetic diversity of source populations and the population genetic structure of a species, to provide a balanced viewpoint for optimal management of endemic taxa under threat of climate-induced range shifts. Given the prospect of continuing habitat deterioration, there are likely to be eventual negative genetic and demographic consequences for *F*. *rostrata* without intervention. As the number of alleles per locus contained within individual populations (*A* = 2.167–3.083) was less than the total across populations (*A* = 4.417), we recommend that *F*. *rostrata* seedlings and cuttings should be exchanged amongst existing populations predicted to maintain suitable habitat under the climate change scenarios modelled in this study. Therefore, as a priority, reintroduction efforts should focus initially on using genetic material from the south to enrich the isolated and genetically depauperate populations in the north, which are predicted to come under increasing stress over the next 50 years due to climate change. However, as each of the two regions contain unique alleles, reciprocal augmentation to increase the diversity among the southern population cluster, in addition to using propagules from the north to a much lesser degree, is advised in the medium term. Future research examining several of *F*. *rostrata*’s co-occurring species with similar life-history and habitat preferences is required to confirm the trends found in this study and further define the genetic architecture and long-term needs of these little-studied ecosystem remnants.

## Supporting information

S1 Table A list of presence records of *F*. *rostrata* used for the species distribution modelling. Sourced from herbarium voucher records [49], online database records [50] and field census records from this study.
(DOCX)

S2 Table Bioclimatic, geological and geomorphological variables explored with the model. (DOCX)

S3 Table Soil types predicted to be suitable for *Fontainea rostrata*. (DOCX)

S4 Table Dominant rock types predicted to be suitable for *Fontainea rostrata* based on the detailed solid geology of Queensland. (DOCX)

S5 Table Coupled Model Intercomparison Project Phase 5 (CMIP5) GCMs used in SIMCLIM v 3.0.0.5 [52]. (DOCX)

S6 Table Predictor variables selected for the species distribution model. Percentage contributions of each predictor variable to the final models and minimum and maximum values or selected class codes of each predictor variable across the 20 presence records are given.
(DOCX)

S7 Table The seven regional ecosystem (RE) types identified by the Queensland Herbarium (2014) to accommodate *Fontainea rostrata*. Approximate area abundance of RE vegetation types within the species’ high-quality habitat is given in km^2^ with percentage contribution to the total in parentheses. Area abundance of vegetation communities other than the seven RE types within the species’ preferred habitat envelope is also given (other RE types and non-remnant).
(DOCX)

S8 Table Allelic frequencies for the nine populations of *Fontainea rostrata* assessed. (DOCX)

S9 Table Summary of population genetic measures for the nine populations (and northern and southern population clusters) of *Fontainea rostrata* with and without nine-loci sub-analyses. Means are shown between horizontal lines.
(DOCX)

S1 Fig Results of Mantel test for correlation (*Rxy*) between genetic and geographic distance matrices in *Fontainea rostrata* across all populations. The probability of *statistical significance (p) based on 999 random permutations is given.
(DOCX)

S2 Fig Regional AMOVA showing the partitioning of variation (*F*_*IT*_) among regions (7%), populations within regions (8%), between individuals (14%), and within populations (71%). (DOCX)

S3 Fig Evanno’s Delta K and proportion of membership (K = 3) within each of the nine *Fontainea rostrata* populations. (DOCX)
