# Supplementary material for: A multidisciplinary approach to inform assisted migration of the restricted rainforest tree, Fontainea rostrata
Source: PLoS One. 2019 Jan 25;14(1):e0210560. doi: 10.1371/journal.pone.0210560 (PMC6347239; doi:10.1371/journal.pone.0210560)
Supplement: S1 Table — Sourced from herbarium voucher records [49], online database records [50] and field census records from this study. (DOCX) [file pone.0210560.s001.docx]

**S1 Table. A list of presence records of *F. rostrata* used for the species distribution modelling.** Sourced from herbarium voucher records [49], online database records [50] and field census records from this study.

| Site | Locality | latitude | longitude | Precision | Datum | Zone | Record | Resource |
| --- | --- | --- | --- | --- | --- | --- | --- | --- |
| 1 | Allen Rd | -26.1187 | 152.6154 | <100 | GDA94 | 56 | 2015 | Field census 2015 |
| 2 | Aural Vale Rd | -26.1022 | 152.7000 | <100 | GDA94 | 56 | 2015 | Field census 2015 |
| 3 | Burns Road | -26.0966 | 152.7568 | <100 | GDA94 | 56 | 2015 | Field census 2015 |
| 4 | Goomboorian | -26.0734 | 152.7761 | <1600 | GDA94 | 56 | 1999 | Herbarium Voucher Records [50] |
| 5 | Gympie | -26.0734 | 152.7261 | <1600 | GDA94 | 56 | 1983 | Atlas of Living Australia [51] |
| 6 | Laurel Rd | -26.1061 | 152.7109 | <100 | GDA94 | 56 | 2015 | Field census 2015 |
| 7 | Laurel Rd | -26.1071 | 152.7113 | <100 | GDA94 | 56 | 2015 | Field census 2015 |
| 8 | Maryborough | -25.6567 | 152.6761 | <1600 | GDA94 | 56 | 1994 | Herbarium Voucher Records [50] |
| 9 | Ormes Rd | -26.1192 | 152.8157 | <100 | GDA94 | 56 | 2015 | Field census 2015 |
| 10 | Puller Rd | -25.7084 | 152.6556 | <100 | GDA94 | 56 | 2015 | Field census 2015 |
| 11 | Teddington Weir | -25.6548 | 152.6708 | <100 | GDA94 | 56 | 1997 | Herbarium Voucher Records [50] |
| 12 | Tinana Creek 1 | -25.7114 | 152.6567 | <100 | GDA94 | 56 | 1991 | Herbarium Voucher Records [50] |
| 13 | Tinana Creek 2 | -25.7105 | 152.6557 | <100 | GDA94 | 56 | 1992 | Herbarium Voucher Records [50] |
| 14 | Tinana Creek 3 | -25.6567 | 152.6594 | <1600 | GDA94 | 56 | 1993 | Herbarium Voucher Records [50] |
| 15 | Tristram Bath Rd | -26.1533 | 152.7842 | <100 | GDA94 | 56 | 2015 | Field census 2015 |
| 16 | Veteran | -26.1234 | 152.7094 | <1600 | GDA94 | 56 | 1992 | Herbarium Voucher Records [50] |
| 17 | Weir Rd 1 | -25.6539 | 152.6695 | <100 | GDA94 | 56 | 2015 | Field census 2015 |
| 18 | Weir Rd 2 | -25.6514 | 152.6694 | <100 | GDA94 | 56 | 2015 | Field census 2015 |
| 19 | Wolvi | -26.1527 | 152.7873 | <100 | GDA94 | 56 | 2007 | Herbarium Voucher Records [50] |
| 20 | Wolvi State Forest | -26.2484 | 152.9177 | <1600 | GDA94 | 56 | 1987 | Herbarium Voucher Records [50] |
