# Supplementary material for: A multidisciplinary approach to inform assisted migration of the restricted rainforest tree, Fontainea rostrata
Source: PLoS One. 2019 Jan 25;14(1):e0210560. doi: 10.1371/journal.pone.0210560 (PMC6347239; doi:10.1371/journal.pone.0210560)
Supplement: S2 Table — (DOCX) [file pone.0210560.s002.docx]

**S2** **Table.** **Bioclimatic, geological and geomorphological variables explored with the model.**

| Data description (ID) | Units | Source/Link |
| --- | --- | --- |
| 1 Annual mean temperature | C˚ | SimCLIM (Climsystems, 2013) |
| 2 Max temperature of warmest month | C˚ |  |
| 3 Min temperature of coldest month | C˚ |  |
| 4 Mean temperature of wettest quarter | C˚ |  |
| 5 Mean temperature of driest quarter | C˚ |  |
| 6 Mean temperature of warmest quarter | C˚ |  |
| 7 Mean temperature of coldest quarter | C˚ |  |
| 8 Annual precipitation | mm |  |
| 9 Precipitation of wettest month | mm |  |
| 10 Precipitation of driest month | mm |  |
| 11 Precipitation of wettest quarter | mm |  |
| 12 Precipitation of driest quarter | mm |  |
| 13 Precipitation of warmest quarter | mm |  |
| 14 Precipitation of coldest quarter | mm |  |
| 15 Soil | Soil classes | (McKenzie *et al*., 2000) |
| 16 Geology | Rock classes | (DNRM, 2011) |
| 17 Proximity to watercourse | Metres | (DNRM, 2000) |
