# Supplementary material for: A multidisciplinary approach to inform assisted migration of the restricted rainforest tree, Fontainea rostrata
Source: PLoS One. 2019 Jan 25;14(1):e0210560. doi: 10.1371/journal.pone.0210560 (PMC6347239; doi:10.1371/journal.pone.0210560)
Supplement: S3 Table — (DOCX) [file pone.0210560.s003.docx]

**S3 Table. Soil types predicted to be suitable for *Fontainea rostrata*** [55].

| Code | Classification | Factual key description |
| --- | --- | --- |
|  |  |  |
| Tb69 | Sodosol | Low rounded hilly terrain on shales and soft sandstones--gentle side slopes to shallow relatively broad valleys: chief soils are hard acidic yellow mottled soils (Dy3.41) and (Dy3.31) of shallow to moderate depth. Associated are shallow to moderately deep (Dr3.41) and (Dr3.31) soils on slopes; (Um2.12) soils on crests; and (Gn2.91, Gn2.94, and Gn2.95) soils with some (Dy2.31) soils in valley flats. Other soil occurrences include (Gn3.42), (Db1.13), and (Um6.21) on andesitic dykes; (Db1.33) on limestone lenses; and a (Dd2.31-Dy3.31) soil complex on siltstones. As mapped, ridges and knolls of red earths (Gn2.14) of unit Mw23 and valleys of unit Mm8 are included |
| Mr6 | Kandosol | Undulating to strongly undulating weakly dissected old coastal plain about 200 ft above sea level with some flat-tops and convex ridge crests above 200 ft, long gentle slopes to shallow open terraced valleys; some high sandstone inselbergs rise abruptly to over 450 ft: chief soils are acid yellow earths (Gn2.24), (Gn2.64), and (Gn2.74) on the broad crests. Associated are (Gn2.94) soils on slopes and creek flats, and (Uc2.2), (Uc2.33), and sometimes (Uc2.35) soils in treeless flat-bottomed valleys. Other soils include: (Gn2.14) on higher ridge crests (above 200 ft) and on the third terrace along major streams; (Gn3.52) on second terrace of major streams; (Dy5.51) and (Dy5.11) with hummocky microrelief along some stream flats; and (Uc2.12) and (Dr2.41) with (Dy2.41) on crests and slopes, respectively, of the inselbergs. As mapped, small areas of mountainous basaltic country with (Gn3.14), (Uf6.32), (Um6.21), and (Gn3. 11) soils are included |
| Pc2 | Chromosol | Hilly to low hilly terrain on phyllites--convex hills with moderate side slopes, small slope fans, and relatively narrow stream valleys: chief soils are hard acid red soils (Dr2.31), (Dr3.31), (Dr2.21), and (Dr2.41) on the slopes. Associated are (Um2.12) soils on hill crests and (Gn3.71) soils on lower slopes and fans. Other soils encountered are: (Um4.1) on hill crests; (Dr3.41) on crests and upper slopes; (Gn3.41) on young fans; (Gn2.64) and (Gn2.94) along stream valleys; (Gn3.74) on benches; and (Gn2.14) on terraces in some localities. |
| Mo9 | Ferrosol | Steep hilly to mountainous land on diorite, moderate to steep slopes, fringing pediment, some rock outcrop, narrow stream valleys: chief soils are neutral red friable earths (Gn3.12) on crests, slopes, and some pediments. Other soils include (Dr4.21), (Dy3.31), and (Gn2.41) on slopes and (Gn2.84), (Gn3.92), (Um1.43), and (Ug5) along stream valleys. |
| Fu6 | Tenosol | Hilly to steep hilly land on shales and cherts; narrow ridge crests with moderate to steep side slopes; narrow valleys; and shallow gravelly soils: chief soils are leached loams (Um2.12) on crests and slopes. Associated are (Um4.1), (Uc2.12), and shallow (Dy3.41) and (Dr3.41) soils. Small areas of many other soils, especially those of unit Fu8, occur throughout. As mapped, there are inclusions of units Mm8 and X13. |
