# Supplementary material for: A multidisciplinary approach to inform assisted migration of the restricted rainforest tree, Fontainea rostrata
Source: PLoS One. 2019 Jan 25;14(1):e0210560. doi: 10.1371/journal.pone.0210560 (PMC6347239; doi:10.1371/journal.pone.0210560)
Supplement: S4 Table — (DOCX) [file pone.0210560.s004.docx]

**S4 Table. Dominant rock types predicted to be suitable for *Fontainea rostrata* based on the detailed solid geology of Queensland** [56].

| Classification code | | Description |
| --- | --- | --- |
| 1 | ALLV | Alluvium |
| 2 | ARMU | Arenite-mudrock |
| 3 | ARNT | Arenite |
| 17 | MSFV | Mixed sedimentary rocks and felsites |
| 25 | SEDS | Sedimentary rock |
