# Supplementary material for: A multidisciplinary approach to inform assisted migration of the restricted rainforest tree, Fontainea rostrata
Source: PLoS One. 2019 Jan 25;14(1):e0210560. doi: 10.1371/journal.pone.0210560 (PMC6347239; doi:10.1371/journal.pone.0210560)
Supplement: S5 Table — (DOCX) [file pone.0210560.s005.docx]

**S5 Table. Coupled Model Intercomparison Project Phase 5 (CMIP5) GCMs used in SIMCLIM v 3.0.0.5 [52]**

| No | Model | Country | Spatial resolution for atmospheric variable (longitude*latitude) | Spatial resolution for ocean variable (longitude*latitude) |
| --- | --- | --- | --- | --- |
| 1 | ACCESS1.3 | Australia | 192*145 | 360*300 |
| 2 | ACCESS1.0 | Australia | 192*145 | 360*300 |
| 3 | BCC-CSM1-1 | China | 128*64 | 360*232 |
| 4 | BCC-CSM1-1-m | China | 320*160 | 360*232 |
| 5 | BNU-ESM | China | 128*64 |  |
| 6 | CanESM2 | Canada | 128*64 | 256*192 |
| 7 | CCSM4 | USA | 288*192 | 320*384 |
| 8 | CESM1-BGC | USA | 288*192 | 320*384 |
| 9 | CESM1-CAM5 | USA | 288*192 | 320*384 |
| 10 | CMCC-CM | Italy | 480*240 | 182*149 |
| 11 | CMCC-CMS | Italy | 192*96 | 182*149 |
| 12 | CNRM-CM5 | France | 256*128 | 362*292 |
| 13 | CSIRO-Mk3-6-0 | Australia | 192*96 | 192*189 |
| 14 | EC-EARTH | Netherlands | 320*160 | 362*292 |
| 15 | FGOALS-g2 | China | 128*60 | 360*196 |
| 16 | FGOALS-s2 | China | 128*108 | 360*196 |
| 17 | GFDL-CM3 | USA | 144*90 | 360*200 |
| 18 | GFDL-ESM2G | USA | 144*90 | 360*210 |
| 19 | GFDL-ESM2M | USA | 144*90 | 360*200 |
| 20 | GISS-E2-H | USA | 144*90 | 144*90 |
| 21 | GISS-E2-H-CC | USA | 144*90 | 144*90 |
| 22 | GISS-E2-R | USA | 144*90 | 288*180 |
| 23 | GISS-E2-R-CC | USA | 144*90 | 288*180 |
| 24 | HADCM3 | UK | 96*73 | 96*73 |
| 25 | HadGEM2-AO | UK | 192*145 | 360*216 |
| 26 | HadGEM2-CC | UK | 192*145 | 360*216 |
| 27 | HadGEM2-ES | UK | 192*145 | 360*216 |
| 28 | INMCM4 | Russia | 180*120 | 360*340 |
| 29 | IPSL-CM5A-LR | France | 96*96 | 182*149 |
| 30 | IPSL-CM5A-MR | France | 144*142 | 182*149 |
| 31 | IPSL-CM5B-LR | France | 96*96 | 182*149 |
| 32 | MIROC4H | Japan | 640*320 | 1280*912 |
| 33 | MIROC5 | Japan | 256*128 | 256*224 |
| 34 | MIROC-ESM | Japan | 128*64 | 256*192 |
| 35 | MIROC-ESM-CHEM | Japan | 128*64 | 256*192 |
| 36 | MPI-ESM-LR | Germany | 192*96 | 256*220 |
| 37 | MPI-ESM-MR | Norway | 192*96 | 802*404 |
| 38 | MRI-CGCM3 | Japan | 320*160 | 360*368 |
| 39 | NorESM1-M | Norway | 144*96 | 320*384 |
| 40 | NorESM1-ME | Norway | 144*96 | 320*384 |
