# Supplementary material for: A multidisciplinary approach to inform assisted migration of the restricted rainforest tree, Fontainea rostrata
Source: PLoS One. 2019 Jan 25;14(1):e0210560. doi: 10.1371/journal.pone.0210560 (PMC6347239; doi:10.1371/journal.pone.0210560)
Supplement: S6 Table — Percentage contributions of each predictor variable to the final models and minimum and maximum values or selected class codes of each predictor variable across the 20 presence records are given. (DOCX) [file pone.0210560.s006.docx]

**S6 Table.** **The six predictor variables selected for the final *Fontainea rostrata* species distribution model.** Percentage contributions of each predictor variable to the final models and minimum and maximum values or selected class codes of each predictor variable across the 20 presence records are given.

| Predictor variable | % contribution | Unit | Max/min (or class codes) | |
| --- | --- | --- | --- | --- |
| Soil | 62.1 | class | Tb69, Mr6, Pc2, Mo9, Fu6 | |
| Precipitation of coldest quarter | 29.5 | mm | 166/245 |  |
| Geology | 5.9 | class | ALLV, ARMU, MSMV, SEDS, ARNT | |
| Mean temperature of warmest quarter | 2.1 | C˚ | 23/24 |  |
| Proximity to watercourse | 0.3 | m | 0/625 |  |
| Mean temperature of coldest quarter | <0.1 | C˚ | 13/15 |  |
