# Supplementary material for: A multidisciplinary approach to inform assisted migration of the restricted rainforest tree, Fontainea rostrata
Source: PLoS One. 2019 Jan 25;14(1):e0210560. doi: 10.1371/journal.pone.0210560 (PMC6347239; doi:10.1371/journal.pone.0210560)
Supplement: S7 Table — Approximate area abundance of RE vegetation types within the species’ high-quality habitat is given in km2 with percentage contribution to the total in parentheses. Area abundance of vegetation communities other than the seven RE types within the species’ preferred habitat envelope is also given (other RE types and non-remnant). (DOCX) [file pone.0210560.s007.docx]

**S7 Table.** **The seven regional ecosystem (RE) types identified by the Queensland Herbarium to accommodate *Fontainea rostrata*.** Approximate area abundance of RE vegetation types within the species’ high quality habitat is given in km^2^ with percentage contribution to the total in parentheses. Area abundance of vegetation communities other than the seven RE types within the species’ high quality habitat is also given (other RE types and non-remnant).

| RE type | Description | Approximate  Area cover |
| --- | --- | --- |
| 12.11.1 | Simple notophyll vine forest often with abundant *Archontophoenix cunninghamiana* (gully vine forest) on metamorphics +/- interbedded volcanics (least concern) | 65km^2^ (8%) |
| 12.11.10 | Notophyll vine forest +/- *Araucaria cunninghamii* on metamorphics +/- interbedded volcanics (least concern) |  |
| 12.11.11 | *Araucarian microphyll* vine forest on metamorphics +/- interbedded volcanics; usually southern half of bioregion (least concern) |  |
| 12.11.16 | *Eucalyptus cloeziana* open forest with vine forest understory on metamorphics +/- interbedded volcanics (endangered) |  |
| 12.12.16 | Notophyll vine forest on Mesozoic to Proterozoic igneous rocks (least concern) |  |
| 12.3.1 | Gallery rainforest (notophyll vine forest) on alluvial plains (endangered) |  |
| 12.3.2 | *Eucalyptus grandis* tall open forest with vine forest understorey on alluvial plains (of concern) |  |
| Other RE types | | 262 km^2^ (30%) |
| Non-remnant (cleared, plantations etc.) | | 538 km^2^ (62%) |
| Total area cover | | 865 km^2^ |
