# Supplementary material for: A multidisciplinary approach to inform assisted migration of the restricted rainforest tree, Fontainea rostrata
Source: PLoS One. 2019 Jan 25;14(1):e0210560. doi: 10.1371/journal.pone.0210560 (PMC6347239; doi:10.1371/journal.pone.0210560)
Supplement: S8 Table — (DOCX) [file pone.0210560.s008.docx]

**S8** **Table.** **Allelic frequencies for the nine populations of *Fontainea rostrata* assessed.**

| **Locus** | **Allele** | **Allen Rd** | **Aural Vale Rd** | **Laurel Rd** | **Ormes Rd** | **Burns Rd** | **Tristram Bath Rd** | **Tahiti Rd** | **Weir Rd** | **Weir Rd 2** |
| --- | --- | --- | --- | --- | --- | --- | --- | --- | --- | --- |
| **FP20** | **N** | 23 | 21 | 24 | 20 | 22 | 26 | 29 | 24 | 22 |
|  | **149** | 1.000 | 1.000 | 1.000 | 1.000 | 1.000 | 0.885 | 1.000 | 1.000 | 1.000 |
|  | **155** | 0.000 | 0.000 | 0.000 | 0.000 | 0.000 | 0.115 | 0.000 | 0.000 | 0.000 |
| **FP21** |  |  |  |  |  |  |  |  |  |  |
|  | **184** | 0.217 | 0.476 | 0.563 | 0.850 | 0.705 | 0.904 | 0.500 | 0.563 | 0.545 |
|  | **186** | 0.348 | 0.071 | 0.042 | 0.025 | 0.273 | 0.000 | 0.328 | 0.229 | 0.182 |
|  | **208** | 0.043 | 0.000 | 0.000 | 0.000 | 0.000 | 0.000 | 0.017 | 0.000 | 0.000 |
|  | **238** | 0.391 | 0.452 | 0.396 | 0.125 | 0.023 | 0.096 | 0.155 | 0.208 | 0.273 |
| **FP24** |  |  |  |  |  |  |  |  |  |  |
|  | **213** | 0.457 | 0.476 | 0.292 | 0.375 | 0.295 | 0.212 | 0.879 | 0.917 | 0.886 |
|  | **217** | 0.152 | 0.071 | 0.542 | 0.025 | 0.023 | 0.096 | 0.000 | 0.000 | 0.045 |
|  | **223** | 0.326 | 0.286 | 0.021 | 0.350 | 0.659 | 0.596 | 0.121 | 0.083 | 0.068 |
|  | **225** | 0.000 | 0.000 | 0.021 | 0.150 | 0.000 | 0.000 | 0.000 | 0.000 | 0.000 |
|  | **227** | 0.022 | 0.000 | 0.000 | 0.000 | 0.000 | 0.019 | 0.000 | 0.000 | 0.000 |
|  | **231** | 0.000 | 0.000 | 0.063 | 0.000 | 0.023 | 0.077 | 0.000 | 0.000 | 0.000 |
|  | **233** | 0.000 | 0.048 | 0.063 | 0.100 | 0.000 | 0.000 | 0.000 | 0.000 | 0.000 |
|  | **235** | 0.022 | 0.119 | 0.000 | 0.000 | 0.000 | 0.000 | 0.000 | 0.000 | 0.000 |
|  | **239** | 0.022 | 0.000 | 0.000 | 0.000 | 0.000 | 0.000 | 0.000 | 0.000 | 0.000 |
| **FP32** |  |  |  |  |  |  |  |  |  |  |
|  | **182** | 0.043 | 0.000 | 0.000 | 0.000 | 0.023 | 0.000 | 0.000 | 0.000 | 0.000 |
|  | **184** | 0.957 | 1.000 | 0.875 | 0.925 | 0.955 | 0.981 | 1.000 | 1.000 | 1.000 |
|  | **190** | 0.000 | 0.000 | 0.042 | 0.050 | 0.023 | 0.019 | 0.000 | 0.000 | 0.000 |
|  | **192** | 0.000 | 0.000 | 0.083 | 0.025 | 0.000 | 0.000 | 0.000 | 0.000 | 0.000 |
| **FP33** |  |  |  |  |  |  |  |  |  |  |
|  | **165** | 0.000 | 0.000 | 0.000 | 0.000 | 0.000 | 0.038 | 0.052 | 0.000 | 0.045 |
|  | **167** | 1.000 | 1.000 | 0.750 | 0.900 | 0.409 | 0.865 | 0.483 | 0.542 | 0.705 |
|  | **169** | 0.000 | 0.000 | 0.250 | 0.100 | 0.591 | 0.096 | 0.466 | 0.458 | 0.205 |
|  | **171** | 0.000 | 0.000 | 0.000 | 0.000 | 0.000 | 0.000 | 0.000 | 0.000 | 0.045 |
| **FP38** |  |  |  |  |  |  |  |  |  |  |
|  | **137** | 0.565 | 0.333 | 0.521 | 0.075 | 0.545 | 0.269 | 0.276 | 0.354 | 0.682 |
|  | **143** | 0.435 | 0.667 | 0.479 | 0.925 | 0.455 | 0.731 | 0.724 | 0.646 | 0.318 |
| **FP39** |  |  |  |  |  |  |  |  |  |  |
|  | **189** | 0.239 | 0.524 | 0.042 | 0.125 | 0.341 | 0.212 | 0.379 | 0.104 | 0.364 |
|  | **201** | 0.565 | 0.405 | 0.667 | 0.850 | 0.545 | 0.788 | 0.259 | 0.688 | 0.545 |
|  | **203** | 0.000 | 0.000 | 0.146 | 0.025 | 0.000 | 0.000 | 0.000 | 0.000 | 0.000 |
|  | **207** | 0.196 | 0.071 | 0.146 | 0.000 | 0.114 | 0.000 | 0.362 | 0.208 | 0.091 |
| **FP40** |  |  |  |  |  |  |  |  |  |  |
|  | **132** | 0.348 | 0.286 | 0.250 | 0.375 | 0.205 | 0.038 | 0.224 | 0.125 | 0.000 |
|  | **134** | 0.000 | 0.024 | 0.146 | 0.000 | 0.045 | 0.000 | 0.017 | 0.000 | 0.000 |
|  | **136** | 0.000 | 0.024 | 0.125 | 0.025 | 0.091 | 0.000 | 0.017 | 0.000 | 0.000 |
|  | **140** | 0.630 | 0.524 | 0.458 | 0.500 | 0.455 | 0.519 | 0.741 | 0.875 | 1.000 |
|  | **142** | 0.000 | 0.071 | 0.000 | 0.100 | 0.205 | 0.442 | 0.000 | 0.000 | 0.000 |
|  | **144** | 0.000 | 0.071 | 0.021 | 0.000 | 0.000 | 0.000 | 0.000 | 0.000 | 0.000 |
|  | **146** | 0.022 | 0.000 | 0.000 | 0.000 | 0.000 | 0.000 | 0.000 | 0.000 | 0.000 |
| **FP41** |  |  |  |  |  |  |  |  |  |  |
|  | **131** | 0.370 | 0.405 | 0.229 | 0.425 | 0.341 | 0.135 | 0.138 | 0.292 | 0.432 |
|  | **155** | 0.630 | 0.595 | 0.771 | 0.575 | 0.659 | 0.865 | 0.862 | 0.708 | 0.568 |
| **FP44** |  |  |  |  |  |  |  |  |  |  |
|  | **108** | 0.000 | 0.167 | 0.125 | 0.000 | 0.114 | 0.173 | 0.000 | 0.042 | 0.045 |
|  | **110** | 1.000 | 0.548 | 0.792 | 1.000 | 0.886 | 0.827 | 0.862 | 0.917 | 0.818 |
|  | **112** | 0.000 | 0.000 | 0.000 | 0.000 | 0.000 | 0.000 | 0.000 | 0.000 | 0.136 |
|  | **114** | 0.000 | 0.286 | 0.083 | 0.000 | 0.000 | 0.000 | 0.138 | 0.042 | 0.000 |
| **FP49** |  |  |  |  |  |  |  |  |  |  |
|  | **163** | 0.000 | 0.000 | 0.000 | 0.100 | 0.000 | 0.154 | 0.000 | 0.000 | 0.000 |
|  | **165** | 0.152 | 0.048 | 0.000 | 0.225 | 0.409 | 0.327 | 0.172 | 0.000 | 0.136 |
|  | **167** | 0.000 | 0.000 | 0.000 | 0.000 | 0.000 | 0.000 | 0.034 | 0.083 | 0.159 |
|  | **169** | 0.848 | 0.952 | 1.000 | 0.675 | 0.591 | 0.519 | 0.793 | 0.917 | 0.705 |
| **FP64** |  |  |  |  |  |  |  |  |  |  |
|  | **99** | 0.000 | 0.024 | 0.000 | 0.000 | 0.114 | 0.000 | 0.000 | 0.000 | 0.000 |
|  | **102** | 0.000 | 0.071 | 0.292 | 0.325 | 0.364 | 0.135 | 0.207 | 0.313 | 0.182 |
|  | **105** | 0.000 | 0.143 | 0.083 | 0.025 | 0.045 | 0.000 | 0.000 | 0.000 | 0.045 |
|  | **108** | 0.391 | 0.119 | 0.521 | 0.275 | 0.182 | 0.404 | 0.552 | 0.563 | 0.773 |
|  | **111** | 0.000 | 0.190 | 0.063 | 0.275 | 0.000 | 0.058 | 0.241 | 0.125 | 0.000 |
|  | **114** | 0.609 | 0.452 | 0.042 | 0.100 | 0.295 | 0.365 | 0.000 | 0.000 | 0.000 |
|  | **123** | 0.000 | 0.000 | 0.000 | 0.000 | 0.000 | 0.038 | 0.000 | 0.000 | 0.000 |
