# Supplementary material for: A multidisciplinary approach to inform assisted migration of the restricted rainforest tree, Fontainea rostrata
Source: PLoS One. 2019 Jan 25;14(1):e0210560. doi: 10.1371/journal.pone.0210560 (PMC6347239; doi:10.1371/journal.pone.0210560)
Supplement: S9 Table — Means are shown between horizontal lines. (DOCX) [file pone.0210560.s009.docx]

**S9 Table. Summary of population genetic measures for the nine populations (and northern and southern population clusters) of *Fontainea rostrata* with and without nine-loci sub-analyses.** Means are shown between horizontal lines.

**Twelve loci**

| **South-western - Mary River Catchment** | | | |
| --- | --- | --- | --- |
| ***A*** | ***H_O_*** | ***H_E_*** | ***F*** |
| 2.417 | 0.286 | 0.348 | 0.225 |
| 2.917 | 0.389 | 0.396 | 0.013 |
| 3.083 | 0.326 | 0.398 | 0.178 |
| 2.806 | 0.334 | 0.381 | 0.139 |
|  |  |  |  |
| **South-eastern - Tinana Creek Catchment** | | | |
| ***A*** | ***H_O_*** | ***H_E_*** | ***F*** |
| 2.833 | 0.283 | 0.333 | 0.194 |
| 2.833 | 0.432 | 0.426 | -0.025 |
| 2.750 | 0.327 | 0.358 | 0.110 |
| 2.806 | 0.347 | 0.372 | 0.093 |
|  |  |  |  |
| **Northern** |  |  |  |
| ***A*** | ***H_O_*** | ***H_E_*** | ***F*** |
| 2.500 | 0.247 | 0.354 | 0.269 |
| 2.167 | 0.264 | 0.307 | 0.206 |
| 2.417 | 0.254 | 0.324 | 0.183 |
| 2.361 | 0.255 | 0.328 | 0.219 |
|  |  |  |  |
| **Southern** |  |  |  |
| ***A*** | ***H_O_*** | ***H_E_*** | ***F*** |
| 2.417 | 0.286 | 0.348 | 0.225 |
| 2.917 | 0.389 | 0.396 | 0.013 |
| 3.083 | 0.326 | 0.398 | 0.178 |
| 2.833 | 0.283 | 0.333 | 0.194 |
| 2.833 | 0.432 | 0.426 | -0.025 |
| 2.750 | 0.327 | 0.358 | 0.110 |
| 2.806 | 0.341 | 0.376 | 0.116 |

**Nine loci – without low *PIC* loci**

| **South-western - Mary River Catchment** | | | |
| --- | --- | --- | --- |
| ***A*** | ***H_O_*** | ***H_E_*** | ***F*** |
| 2.778 | 0.382 | 0.455 | 0.114 |
| 3.556 | 0.519 | 0.528 | 0.013 |
| 3.444 | 0.403 | 0.464 | 0.103 |
| 3.259 | 0.434 | 0.482 | 0.076 |
|  |  |  |  |
| **South-eastern - Tinana Creek Catchment** | | | |
| ***A*** | ***H_O_*** | ***H_E_*** | ***F*** |
| 3.111 | 0.361 | 0.408 | 0.111 |
| 3.111 | 0.556 | 0.505 | -0.117 |
| 2.889 | 0.393 | 0.424 | 0.106 |
| 3.037 | 0.437 | 0.445 | 0.033 |
|  |  |  |  |
| **Northern** |  |  |  |
| ***A*** | ***H_O_*** | ***H_E_*** | ***F*** |
| 2.778 | 0.299 | 0.411 | 0.244 |
| 2.444 | 0.324 | 0.354 | 0.173 |
| 2.556 | 0.323 | 0.381 | 0.105 |
| 2.593 | 0.315 | 0.382 | 0.174 |
|  |  |  |  |
| **Southern** |  |  |  |
| ***A*** | ***H_O_*** | ***H_E_*** | ***F*** |
| 2.778 | 0.382 | 0.455 | 0.114 |
| 3.556 | 0.519 | 0.528 | 0.013 |
| 3.444 | 0.403 | 0.464 | 0.103 |
| 3.111 | 0.361 | 0.408 | 0.111 |
| 3.111 | 0.556 | 0.505 | -0.117 |
| 2.889 | 0.393 | 0.424 | 0.106 |
| 3.148 | 0.435 | 0.464 | 0.055 |

**Nine loci – without low *N_A_* loci**

| **South-western - Mary River Catchment** | | | |
| --- | --- | --- | --- |
| ***A*** | ***H_O_*** | ***H_E_*** | ***F*** |
| 2.667 | 0.290 | 0.358 | 0.194 |
| 3.333 | 0.418 | 0.425 | 0.007 |
| 3.556 | 0.361 | 0.436 | 0.151 |
| 3.185 | 0.356 | 0.406 | 0.117 |
|  |  |  |  |
| **South-eastern - Tinana Creek Catchment** | | | |
| ***A*** | ***H_O_*** | ***H_E_*** | ***F*** |
| 3.222 | 0.322 | 0.374 | 0.192 |
| 3.222 | 0.449 | 0.463 | 0.018 |
| 3.000 | 0.355 | 0.386 | 0.133 |
| 3.148 | 0.375 | 0.407 | 0.114 |
|  |  |  |  |
| **Northern** |  |  |  |
| ***A*** | ***H_O_*** | ***H_E_*** | ***F*** |
| 2.778 | 0.268 | 0.401 | 0.282 |
| 2.333 | 0.264 | 0.312 | 0.209 |
| 2.667 | 0.232 | 0.329 | 0.193 |
| 2.593 | 0.255 | 0.347 | 0.228 |
|  |  |  |  |
| **Southern** |  |  |  |
| ***A*** | ***H_O_*** | ***H_E_*** | ***F*** |
| 2.667 | 0.290 | 0.358 | 0.194 |
| 3.333 | 0.418 | 0.425 | 0.007 |
| 3.556 | 0.361 | 0.436 | 0.151 |
| 3.222 | 0.322 | 0.374 | 0.192 |
| 3.222 | 0.449 | 0.463 | 0.018 |
| 3.000 | 0.355 | 0.386 | 0.133 |
| 3.167 | 0.366 | 0.407 | 0.116 |

**Nine loci – without out of trend *F* loci**

| **South-western - Mary River Catchment** | | | |
| --- | --- | --- | --- |
| ***A*** | ***H_O_*** | ***H_E_*** | ***F*** |
| 2.111 | 0.329 | 0.337 | 0.086 |
| 2.556 | 0.423 | 0.383 | -0.064 |
| 2.667 | 0.352 | 0.344 | 0.003 |
| 2.444 | 0.368 | 0.355 | 0.008 |
|  |  |  |  |
| **South-eastern - Tinana Creek Catchment** | | | |
| ***A*** | ***H_O_*** | ***H_E_*** | ***F*** |
| 2.556 | 0.261 | 0.279 | 0.056 |
| 2.556 | 0.475 | 0.384 | -0.186 |
| 2.444 | 0.325 | 0.327 | 0.045 |
| 2.519 | 0.354 | 0.330 | -0.029 |
|  |  |  |  |
| **Northern** |  |  |  |
| ***A*** | ***H_O_*** | ***H_E_*** | ***F*** |
| 2.333 | 0.272 | 0.343 | 0.131 |
| 2.222 | 0.315 | 0.312 | 0.012 |
| 2.333 | 0.308 | 0.358 | 0.067 |
| 2.296 | 0.298 | 0.338 | 0.070 |
|  |  |  |  |
| **Southern** |  |  |  |
| ***A*** | ***H_O_*** | ***H_E_*** | ***F*** |
| 2.111 | 0.329 | 0.337 | 0.086 |
| 2.556 | 0.423 | 0.383 | -0.064 |
| 2.667 | 0.352 | 0.344 | 0.003 |
| 2.556 | 0.261 | 0.279 | 0.056 |
| 2.556 | 0.475 | 0.384 | -0.186 |
| 2.444 | 0.325 | 0.327 | 0.045 |
| 2.481 | 0.361 | 0.342 | -0.010 |
