# Supplementary material for: A multidisciplinary approach to inform assisted migration of the restricted rainforest tree, Fontainea rostrata
Source: PLoS One. 2019 Jan 25;14(1):e0210560. doi: 10.1371/journal.pone.0210560 (PMC6347239; doi:10.1371/journal.pone.0210560)
Supplement: S1 Fig — The probability of *statistical significance (p) based on 999 random permutations is given. (DOCX) [file pone.0210560.s010.docx]

**S1 Fig. Results of Mantel test for correlation (*Rxy*) between genetic and geographic distance matrices in *Fontainea rostrata* across all populations.** The probability of *statistical significance (*p*) based on 999 random permutations is given.

| ***Rxy*** | ***p*** |
| --- | --- |
| 0.174* | 0.010 |
