# Supplementary material for: A multidisciplinary approach to inform assisted migration of the restricted rainforest tree, Fontainea rostrata
Source: PLoS One. 2019 Jan 25;14(1):e0210560. doi: 10.1371/journal.pone.0210560 (PMC6347239; doi:10.1371/journal.pone.0210560)
Supplement: S2 Fig — (DOCX) [file pone.0210560.s011.docx]

**S2 Fig. Regional AMOVA showing the partitioning of variation (*F_IT_*) among regions (7%), populations within regions (8%), between individuals (14%), and within populations (71%).**

| **Summary AMOVA Table** | |  |  |  |  |
| --- | --- | --- | --- | --- | --- |
|  |  |  |  |  |  |
| **Source** | **df** | **SS** | **MS** | **Est. Var.** | **%** |
| **Among Regions** | 2 | 79.685 | 39.843 | 0.193 | 7% |
| **Among Pops** | 6 | 75.224 | 12.537 | 0.214 | 8% |
| **Among Indiv** | 202 | 519.382 | 2.571 | 0.357 | 14% |
| **Within Indiv** | 211 | 392.000 | 1.858 | 1.858 | 71% |
| **Total** | 421 | 1066.291 |  | 2.621 | 100% |
|  |  |  |  |  |  |
| **F-Statistics** | **Value** | **P(rand >= data)** | |  |  |
| **Frt** | 0.074 | 0.001 |  |  |  |
| **Fsr** | 0.088 | 0.001 |  |  |  |
| **Fst** | 0.155 | 0.001 |  |  |  |
| **Fis** | 0.161 | 0.001 |  |  |  |
| **Fit** | 0.291 | 0.001 |  |  |  |
|  |  |  |  |  |  |
| **Frt max** | 0.595 |  |  |  |  |
| **F'rt** | 0.124 |  |  |  |  |
|  |  |  |  |  |  |
| **Fsr max** | 0.631 |  |  |  |  |
| **F'sr** | 0.139 |  |  |  |  |
|  |  |  |  |  |  |
| **Nm** | 1.360 |  |  |  |  |
|  |  |  |  |  |  |
