# Supplementary material for: A multidisciplinary approach to inform assisted migration of the restricted rainforest tree, Fontainea rostrata
Source: PLoS One. 2019 Jan 25;14(1):e0210560. doi: 10.1371/journal.pone.0210560 (PMC6347239; doi:10.1371/journal.pone.0210560)
Supplement: S3 Fig — (DOCX) [file pone.0210560.s012.docx]

**S3 Table. Evanno’s *Delta K* and proportional of membership (*K* = 3) within each of the nine *Fontainea rostrata* populations and population clusters.**

**
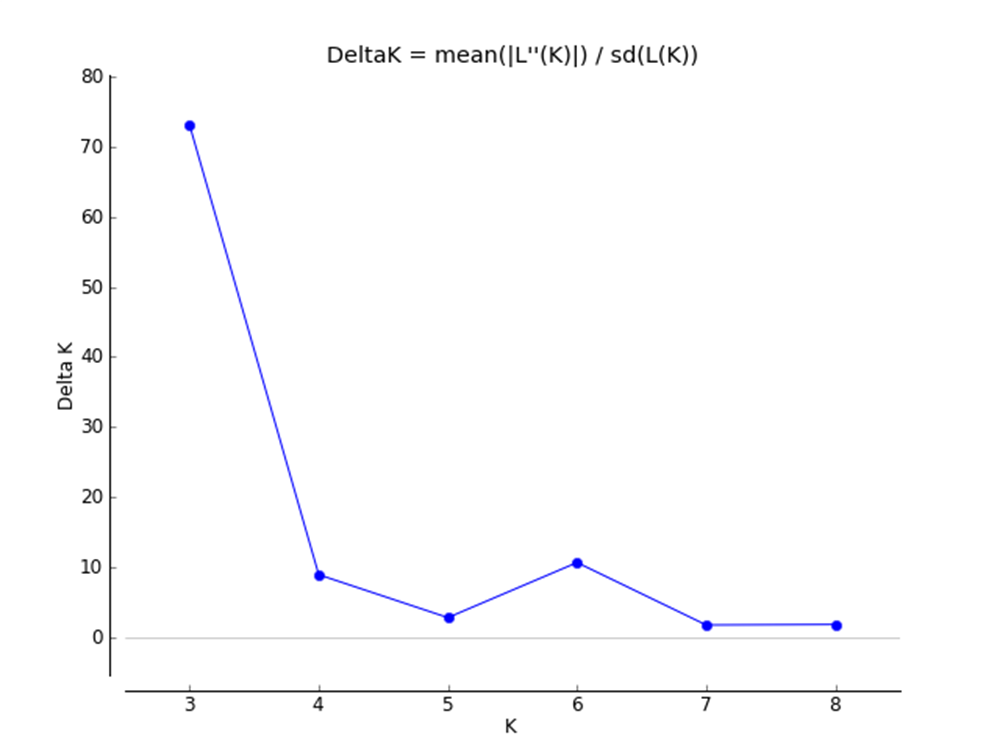
**


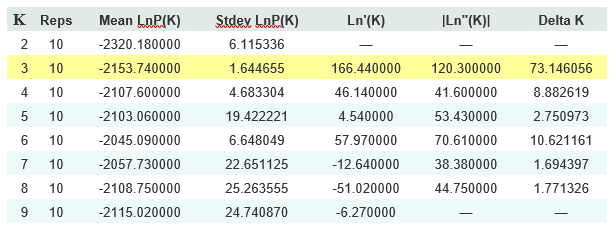


| **Population** | **1** | **2** | **3** |
| --- | --- | --- | --- |
| **Allen Rd - S** | 0.18 | 0.48 | 0.34 |
| **Aural Vale Rd - S** | 0.18 | 0.49 | 0.33 |
| **Laurel Rd - S** | 0.23 | 0.23 | 0.53 |
|  |  |  |  |
| **Ormes Rd - S** | 0.51 | 0.08 | 0.41 |
| **Burns Rd - S** | 0.43 | 0.24 | 0.34 |
| **Tristram Bath Rd - S** | 0.80 | 0.14 | 0.06 |
|  |  |  |  |
| **Tahiti Rd - N** | 0.14 | 0.59 | 0.28 |
| **Weir Rd - N** | 0.30 | 0.55 | 0.15 |
| **Weir Rd 2 - N** | 0.28 | 0.60 | 0.12 |
|  |  |  |  |
